# *In silico* characterization of chitin deacetylase genes in the *Diaphorina citri* genome

**DOI:** 10.46471/gigabyte.25

**Published:** 2021-06-11

**Authors:** Sherry Miller, Teresa D. Shippy, Blessy Tamayo, Prashant S. Hosmani, Mirella Flores-Gonzalez, Lukas A. Mueller, Wayne B. Hunter, Susan J. Brown, Tom D’Elia, Surya Saha

**Affiliations:** ^1^ Division of Biology, Kansas State University, Manhattan, KS 66506, USA; ^2^ Allen County Community College, Burlingame, KS 66413, USA; ^3^ Indian River State College, Fort Pierce, FL 34981, USA; ^4^ Boyce Thompson Institute, Ithaca, NY 14853, USA; ^5^ USDA-ARS, U.S. Horticultural Research Laboratory, Fort Pierce, FL 34945, USA; ^6^ Animal and Comparative Biomedical Sciences, University of Arizona, Tucson, AZ 85721, USA

## Abstract

Chitin deacetylases (CDAs) are one of the least understood components of insect chitin metabolism. The partial deacetylation of chitin polymers appears to be important for the proper formation of higher order chitin structures, such as long fibers and bundles, which contribute to the integrity of the insect exoskeleton and other structures. Some CDAs may also be involved in bacterial defense. Here, we report the manual annotation of four CDA genes from the Asian citrus psyllid, *Diaphorina citri*, laying the groundwork for future study of these genes.

## Data Description

### Introduction

Chitin deacetylases (CDAs) are metalloenzymes that partially deacetylate chitin polymers [1]. CDA activity in insects was first reported in the cabbage looper *Trichoplusia ni* [2]. In *Drosophila melanogaster*, several CDAs are involved in tracheal development [3, 4]. More recently, genomic and phylogenetic studies have shown that CDAs are present widely in insects and can be classified into five different groups [5, 6]. Most holometabolous insects have at least one representative of each of the five CDA groups, while the hemimetabolous insects that have been examined lack group II and group V genes [6]. The exact role of insect CDAs is not well understood, but they may play a role in organization of chitin molecules into higher order structures [7].

### Context

Loss of function experiments indicate that some CDAs are essential for growth and development, making them a potential target for insect pest control [6, 8–12]. Here we describe the chitin deacetylase gene family in the Asian citrus psyllid, *Diaphorina citri* (Hemiptera: Liviidae; NCBI:txid121845). *D. citri* is the vector for *Candidatus* Liberibacter asiaticus (*C*Las), which is responsible for the global outbreak of Huanglongbing (citrus greening) disease. We identified four chitin deacetylase genes in the *D. citri* v3 genome, three of which have multiple isoforms. As in other hemipterans, only groups I, III and IV are represented [6].

**Figure 1.**
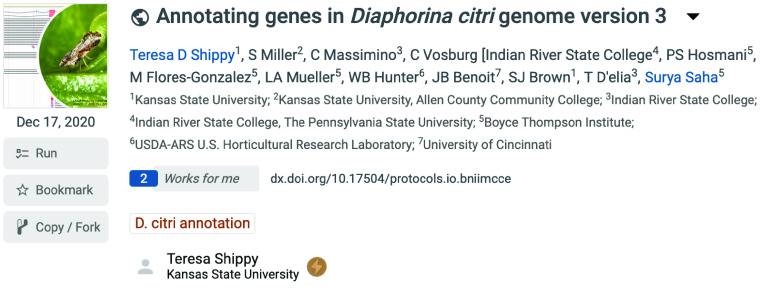
Protocols.io protocol outlining the annotation process of the *D. citri* genome [16]. https://www.protocols.io/widgets/doi?uri=dx.doi.org/10.17504/protocols.io.bniimcce

## Methods

Chitin deacetylase genes in *D. citri* genome v3 [13] were identified by BLAST (NCBI BLAST, RRID:SCR_004870) search of *D. citri* sequences with chitin deacetylase orthologs from other insects. Orthology was confirmed by reciprocal BLAST of the National Center for Biotechnology Information (NCBI) non-redundant protein database [14]. Genes were manually annotated in Apollo 2.1.0 (RRID:SCR_001936) [15] using available evidence, including RNA-seq reads, Iso-seq transcripts and *de novo*-assembled transcripts. A more detailed annotation protocol is available at protocols.io (Figure 1) [16].

Domain analysis was performed with InterPro (InterPro, RRID:SCR_006695) [17]. Multiple alignments were performed using CLUSTALW (RRID:SCR_002909) [18] within MEGA X (MEGA software, RRID:SCR_000667) [19] followed by phylogenetic tree construction using the neighbor-joining method in MEGA X. Table 1 contains a list of orthologs used in the phylogenetic analysis. Expression data from the Citrus Greening Expression Network (CGEN) [20] (Table 2) was visualized using the pheatmap (pheatmap, RRID:SCR_016418) package of R (R Project for Statistical Computing, RRID:SCR_001905) [21, 22] or Microsoft Excel (Microsoft Excel, RRID:SCR_016137).

**Table 1:** Orthologs used in phylogenetic analysis.

Species	Accession	Name in NCBI	Name in Tree
*Nilaparvata lugens*	AJQ20732.1	chitin deacetylase 1	Nl CDA1
*Nilaparvata lugens*	AJQ20733.1	chitin deacetylase 2	Nl CDA2
*Nilaparvata lugens*	AJQ20734.1	chitin deacetylase 3	Nl CDA3
*Nilaparvata lugens*	AJQ20735.1	chitin deacetylase 4	Nl CDA4
*Tribolium castaneum*	NP_001095946.1	chitin deacetylase 1 precursor	Tc CDA1
*Tribolium castaneum*	NP_001116303.1	chitin deacetylase 2 isoform B precursor	Tc CDA2
*Tribolium castaneum*	NP_001104011.1	chitin deacetylase 3 precursor	Tc CDA3
*Tribolium castaneum*	NP_001103903.1	chitin deacetylase 4 precursor	Tc CDA4
*Tribolium castaneum*	NP_001103739.1	chitin deacetylase 5 isoform A precursor	Tc CDA5
*Tribolium castaneum*	NP_001103905.1	chitin deacetylase 6 precursor	Tc CDA6
*Tribolium castaneum*	NP_001104012.1	chitin deacetylase 7 precursor	Tc CDA7
*Tribolium castaneum*	NP_001103906.1	chitin deacetylase 8 precursor	Tc CDA8
*Tribolium castaneum*	NP_001103904.1	chitin deacetylase 9 precursor	Tc CDA9
*Drosophila melanogaster*	NP_001262062.1	serpentine, isoform C	Dm Serp
*Drosophila melanogaster*	NP_730442.2	vermiform, isoform G	Dm verm
*Drosophila melanogaster*	NP_609806.1	ChLD3	Dm ChLD3
*Drosophila melanogaster*	NP_728468.1	chitin deacetylase-like 4	Dm Cda4
*Drosophila melanogaster*	NP_001245808.1	chitin deacetylase-like 5, isoform I	Dm Cda5
*Drosophila melanogaster*	NP_001286519.1	chitin deacetylase-like 9, isoform B	Dm Cda9

Species, accession number, full name and abbreviated name are provided for all orthologs used in phylogenetic analysis.

**Table 2:** TPM expression values.

Gene ID	Dcitr04g03590.1.1	Dcitr04g03590.1.2	Dcitr04g03540.1.1	Dcitr04g03540.1.2	Dcitr02g03950.1.1	Dcitr01g12310.1.1	Dcitr01g12310.1.2	Dcitr01g12310.1.3	Dcitr01g12310.1.4	Dcitr01g12310.1.5
Gene/Transcript Name	CDA1-RB	CDA1-RA	CDA2-RB	CDA2-RA	CDA4	CDA5-RD	CDA5-RC partial	CDA5-RA partial	CDA5-RE	CDA5-RB partial
Egg *Citrus macrophylla* *C*Las− Whole body	56.64	415.8	4.89	22.79	423.69	16.46	0	0	6.95	9.07
Nymph *Citrus medica* *C*Las+ Low infection Whole body	67.1	576.33	111.58	271.48	571.7	50.9	16.28	0	108.52	2.93
Nymph *Citrus sinensis* *C*Las+ High infection Whole body	71.33	513.82	100.65	247.03	374.09	78.16	3.45	0	145.78	3.22
Nymph *C. sinensis* *C*Las− Whole body	78.22	533.18	36.7	242.93	453.44	31.57	0	0	77.51	1.43
Nymph *C. macrophylla* *C*Las− Whole body	63.26	398.54	5.1	15.5	564.13	66.92	0	0	74.46	9.88
Nymph *Citrus* spp. *C*Las− Whole body	118.31	171.29	2.5	11.24	822.56	15.1	3.36	37.94	41.04	7.32
Nymph *Citrus* spp. *C*Las+ Whole body	58.83	343.75	0.36	8.66	260.62	13.61	0	33.96	6.56	8.04
Adult *C. medica* *C*Las− Gut	0.24	0.02	0	0.12	0.27	0	0	0.07	0.3	0
Adult *C. medica* *C*Las+ Gut	0.59	0.09	0.01	0.32	0.54	0.02	0	0.05	0.4	0
Adult *C. medica* *C*Las+ High infection Whole body	40.95	78.19	42.05	40.72	77.88	15	0	0	22.22	0.12
Adult *C. medica* *C*Las+ Low infection Whole body	35.92	94.71	97.78	9.81	99.09	9.59	0	0	27.89	0.07
Adult *C. medica* *C*Las− Whole body	46.28	171.94	125.69	78.25	193.02	29.12	4.81	0	48.75	0.2
Adult *C. macrophylla* *C*Las− Whole body	5.48	16.96	0	0.53	15.86	2.51	0	0	3.67	0
Adult *Citrus* spp. *C*Las− Whole body	14.83	0	0	1.41	66.18	2.36	0	0.39	1.78	0
Adult *Citrus* spp. *C*Las+ Whole body	8.49	9.2	0	3.78	9.23	0.66	1.12	1.98	2.62	0
Adult *Citrus* spp. *C*Las− midgut	0.73	0	0.03	0.5	3.13	0	0	0	0.17	0
Adult *Citrus* spp. *C*Las+ midgut	3.36	5.45	0.02	0.07	11.56	0.13	0.01	0.12	1.02	0
Adult *Citrus reticulata* *C*Las− Female abdomen	7.34	6.88	0	0.48	8.36	0.57	0	0	1.09	0
Adult *C. reticulata* *C*Las− Female antennae	9.65	31.14	0.14	0.75	30.31	1.7	0	0	2.81	0
Adult *C. reticulata* *C*Las− Female head	13.95	12.73	0.54	0.55	20.44	0	0	0.76	2.96	0
Adult *C. reticulata* *C*Las− Female leg	17.46	4.67	0.01	6.6	12.63	0	0	1.16	1.7	0.48
Adult *C. reticulata* *C*Las− Female terminal abdomen	14.73	36.64	0.08	1.27	16.78	0.53	0.08	0	3.73	0.76
Adult *C. reticulata* *C*Las− Female thorax	15.3	7.91	0.04	0.03	15.29	2.53	0	0.2	0.97	0
Adult *C. reticulata* *C*Las− Male abdomen	8.81	2.95	0	2.95	15.63	0.79	0	0.93	0.73	0
Adult *C. reticulata* *C*Las− Male antennae	11.9	36.52	0.79	2.06	30.78	2.14	0	0	4.56	0.13
Adult *C. reticulata* *C*Las− Male head	10.15	17.32	0.45	0.43	28.15	1.21	0	0	2.4	1.12
Adult *C. reticulata* *C*Las− Male leg	17.7	4.13	0.02	6.26	13.4	0.88	0	0.09	2.19	0.41
Adult *C. reticulata* *C*Las− Male terminal abdomen	17.41	20.3	1.29	0.75	25.87	1.9	0	0	5.19	0.16
Adult *C. reticulata* *C*Las− Male thorax	16.31	8.55	0	1.62	17.42	1.65	0	0	4.11	0.32
Adult *C. reticulata* *C*Las− Female antennae [23]	14.37	16.64	0.06	5.52	18.05	0.24	0.04	1.14	1.47	0
Adult *C. reticulata* *C*Las− Female terminal abdomen [23]	5.83	11.74	0	2.95	4.61	0.58	0	0.14	0.48	0.12
Adult *C. reticulata* *C*Las− Male antennae [23]	8.22	26.46	0.91	0.73	17.65	0.3	0	0	5.71	2.59
Adult *C. reticulata* *C*Las− Male terminal abdomen [23]	11.37	6.91	0	3.67	12.05	0.97	0.09	0	2.68	0.1

**Figure 2.**
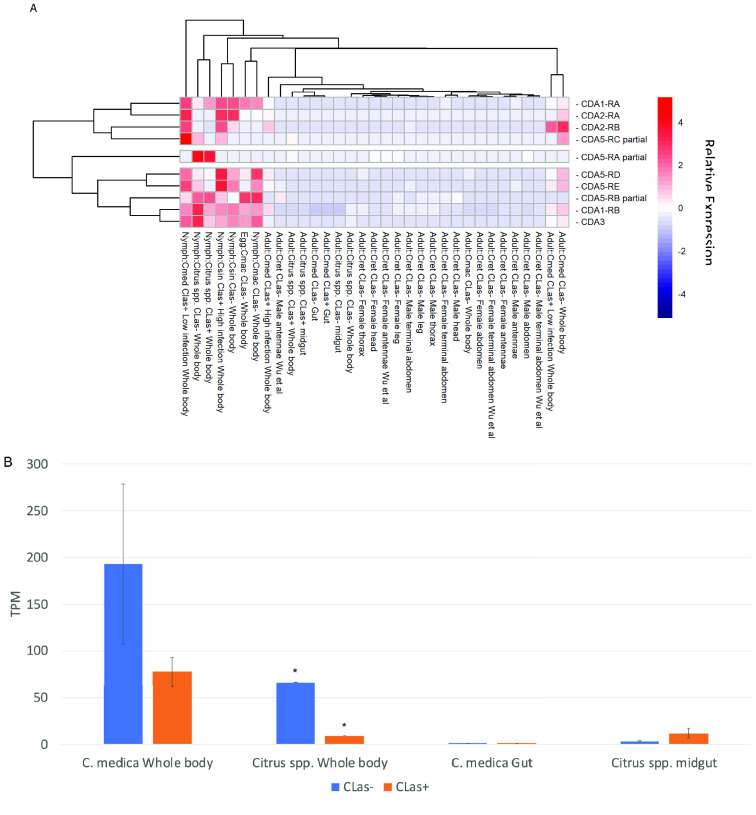
Expression of *D. citri* chitin deacetylase genes. (A) Heatmap displaying relative expression levels of all annotated chitin deacetylase genes in RNA-seq datasets from various life stages, tissues and *C*Las infection states. Expression levels were obtained from CGEN [20] and are reported in Table 2. The heatmap is scaled by row. (B) Expression levels (transcripts per million, TPM) of CDA3 (Dcitr02g03950.1.1) in tissues from *C*Las+ and *C*Las− psyllids fed on two different types of citrus plants. Standard error bars are shown for all expression values except those marked with an asterisk (*), which had only one replicate. Expression levels were obtained from CGEN [20].

Expression levels (transcripts per million, TPM) for annotated chitin deacetylase transcripts from available RNA-seq experiments used for Figure 2. All data are publicly available and were obtained from CGEN [20]. Developmental stage, citrus host, *C*Las (*Candidatus* Liberibacter asiaticus) infection status and tissue of each sample are provided in the first column.

## Data validation and quality control

Chitin deacetylase genes in the *D. citri* v3 genome [13] were identified and manually annotated as described below. These genes were classified following the precedents established in other insects [5, 6].

### Group I chitin deacetylases

Most insects have two group I genes named *CDA1* and *CDA2* (Table 3). The proteins encoded by these genes have an N-terminal chitin-binding domain (ChBD), a low-density lipoprotein receptor class A domain (LDLa), and a deacetylase catalytic domain [5]. RNA interference (RNAi) of group I CDAs in a variety of insects suggests that loss of function of CDA1 or CDA2 can result in lethality and therefore these genes could be potential targets for pest control methods [6–12]. Recent experiments in *Tribolium* suggest that TcCDA1 and TcCDA2 are required for organization of chitin into longer fibers that are important for cuticular strength [7].

**Table 3:** Estimated number of chitin deacetylase homologs.

**Species**	**Group I**	**Group II**	**Group III**	**Group IV**	**Group V**	**Total**
*D. melanogaster*	2*	1	1	1*	1	6
*A. gambiae*	2*	1	1	1	0	5
*T. castaneum*	2*	1	1	1*	4	9
*B. mori*	2*	1	1	1	3	8
*A. mellifera*	2*	1	1	1*	0	5
*N. vitripennis*	2	1	1	1	0	5
*R. prolixus*	2	0	1	1	0	4
*A. pisum*	2	0	1	1	0	4
*N. lugens*	2	0	1	1	0	4
*D. citri*	2*	0	1	1*	0	4

*D. citri* gene numbers were determined based on annotation of the *D. citri* genome v3. All other ortholog numbers were obtained from published sources [5, 6, 24–32]. An asterisk (*) indicates that isoforms have been found for at least one member of the group in that organism.

**Figure 3.**
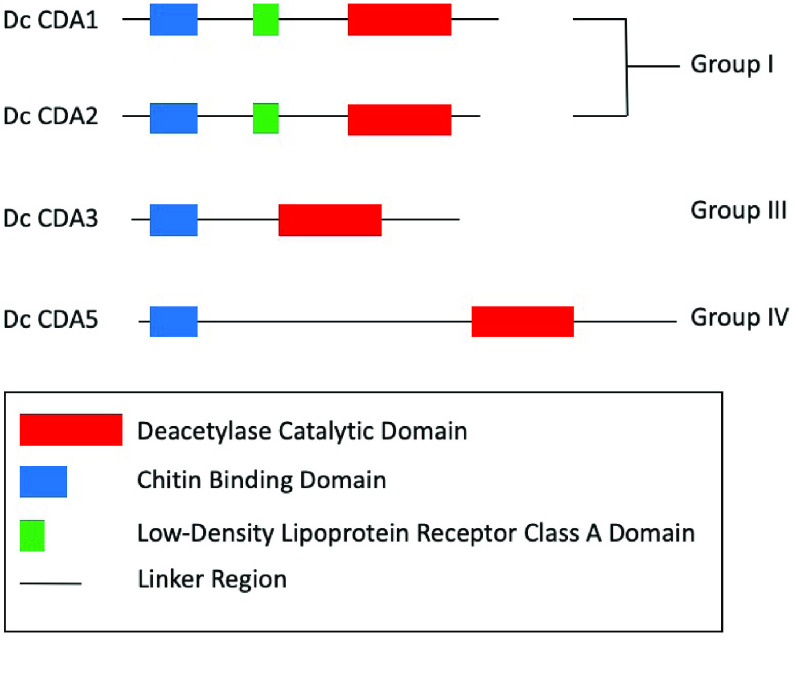
Chitin deacetylase domain organization in *D. citri*. Chitin deacetylases are categorized by group based on phylogenetic analysis, sequence similarity, and domain organization. *D. citri* domain analysis was performed using InterPro [17]. CDA5 is represented by the protein encoded by the de novo-assembled transcript MCOT06229.1.CO [34] because a small portion of the *CDA5* gene is missing from the v3 genome assembly.

As expected, we identified two group I genes in *D. citri*, which we named *CDA1* and *CDA2*. Both genes encode proteins with the typical group I domain structure (Figure 3). We identified two isoforms each for *D. citri CDA1* and *CDA2* (Table 4). *CDA2* has previously been shown to have multiple isoforms in several holometabolous insect species, with the transcripts differing only in the use of one alternative exon [5, 10, 12]. This gene structure is conserved in *D. citri CDA2* with alternate exons 3a and 3b. The two *D. citri CDA1* isoforms differ in the presence or absence of a 24-bp exon upstream of the last exon. Expression data from RNA-seq datasets available through CGEN [20] suggest that, in general, expression of CDA1 and CDA2 is higher in nymphs and eggs than in adults (Figure 2A).

**Figure 4.**
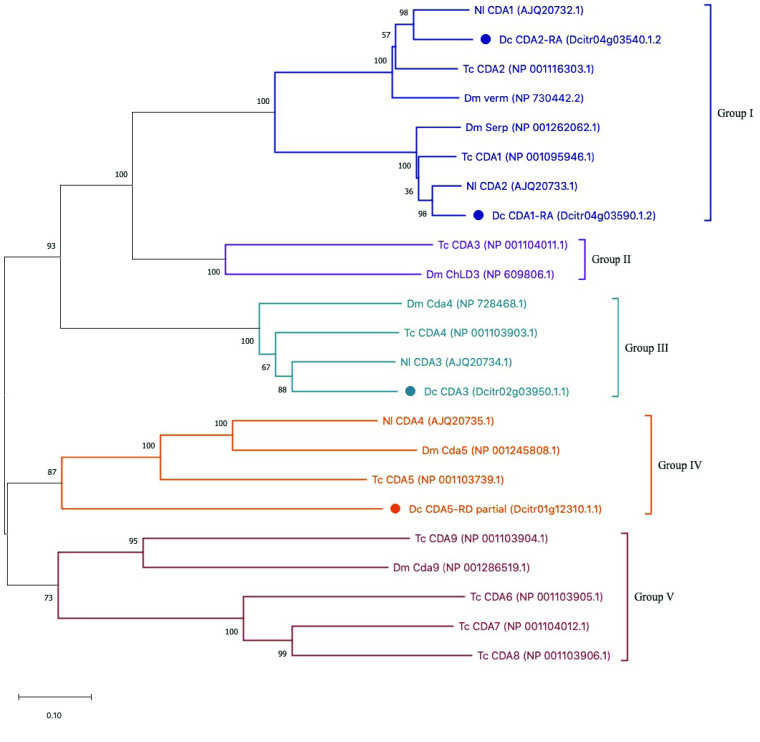
Phylogenetic tree of chitin deacetylase family members. ClustalW was used to perform the multiple sequence alignment. The tree was constructed with MEGA X software [19] using neighbor-joining analysis with 100 bootstrap replications. *Drosophila melanogaster* (Dm), *Tribolium castaneum* (Tc), *Nilaparvata lugens* (Nl), and *Diaphorina citri* (Dc). *D. citri* protein branches are marked with a circle. Colors delineate established chitin deacetylase groups.

**Table 4:** Chitin deacetylase genes in *D. citri*.

Gene/ Isoform	OGSv3 ID	Gene model	Evidence supporting annotation			
		Complete	MCOT	Iso-Seq	RNA-Seq	Ortholog
CDA1 (Group I)	Dcitr04g03590.1.1	X	MCOT00151.2.CO	X	X	X
	Dcitr04g03590.1.2		MCOT00151.1.CO			
CDA2 (Group I)	Dcitr04g03540.1.1	X		X	X	X
	Dcitr04g03540.1.2					
CDA3 (Group III)	Dcitr02g03950.1.1	X	MCOT04789.0.CT	X		X
CDA5 (Group IV)	Dcitr01g12310.1.1		MCOT14896.0.CT	X	X	X
	Dcitr01g12310.1.2		MCOT06229.1.CO			
	Dcitr01g12310.1.3		MCOT06229.3.CO			
	Dcitr01g12310.1.4		MCOT19482.0.CT			
	Dcitr01g12310.1.5		MCOT06229.2.CO			

MCOT: MAKER (MAKER, RRID:SCR_005309), Cufflinks (Cufflinks, RRID:SCR_014597), Oases (Oases, RRID:SCR_011896), Trinity (Trinity, RRID:SCR_013048) pipeline. Each manually annotated gene has been assigned an OGSv3.0 gene identifier. Genes not marked as complete were only able to be annotated as partial gene models. Evidence types used for manual annotation of each gene are indicated. More information on these evidence sources is available in [16].

In *Drosophila* and *Tribolium*, the *CDA1* and *CDA2* orthologs are adjacent to one another in the genome [3, 5] on chromosomes 3 and 5, respectively. The conserved clustering of these genes suggests there may be evolutionary constraint on their physical location. We found that the *D. citri CDA1* and *CDA2* orthologs are also adjacent to one another on chromosome 4. In the *D. citri* v3 genome, these genes are separated by approximately 50 kilobase pairs (Kb), although this distance appears to be inflated by falsely duplicated fragments of both genes in this assembly.

### Group III chitin deacetylases

We identified one group III CDA in the *D. citri* v3 genome (Tables 3 and 4). This gene has been previously described and was named *CDA3* because of its orthology to *Nilaparvata lugens CDA3* [33]. Like group III CDAs in other insects, *D. citri* CDA3 contains a ChBD and catalytic domain but lacks the low-density lipoprotein receptor class A (LDLa) domain found in group I CDAs (Figure 3). Improvements in the genome assembly mean that our curated CDA3 model from genome v3 has additional 3*′* sequence than the previously reported model (GenBank accession number XM_008481889.1), which was based on genome v1.1 [33, 34]. The resulting predicted protein is almost 50 amino acids longer, with additional conserved sequence at the C-terminus.

Yu *et al.* [33] reported that RNAi knockdown of *CDA3* had no effect on molting or wing development. Instead, their results implicated *CDA3* in the *D. citri* bacterial immune response. Recombinant CDA3 showed antibacterial activity against gram-positive bacteria, but had no effect on gram-negative bacteria. Moreover, injection of either *Escherichia coli* (gram-negative) or *Staphylococcus aureus* (gram-positive) bacteria into *D. citri* increased *CDA3* expression in the midgut and decreased its expression in the fat body, although the timeline of these effects is not certain. To determine whether infection by *C*Las, a gram-negative bacterium, might also affect *CDA3* expression, we used CGEN [20] to compare expression of *D. citri CDA3* in RNA-seq datasets from *C*Las+ and *C*Las− guts [35], midguts [36] and whole bodies ([37] and NCBI BioProject PRJNA609978). *CDA3* expression was lower in *C*Las+ versus *C*Las− whole body tissue in data from two different RNA-Seq experiments (Figure 2B). Expression of *CDA3* in midgut and gut tissues was low in all samples but there was a slight increase in *C*Las+ versus *C*Las− midgut expression (Figure 2B). While the significance of these expression differences is not clear, they may warrant further investigation.

### Group IV chitin deacetylases

Most insects examined to date have one group IV CDA, typically called *CDA5* (*CDA4* in *N. lugens*) (Table 3). CDA5 has been shown to have multiple isoforms in *Tribolium* and *Drosophila* [5]. Consistent with these observations, we identified and annotated five different isoforms of CDA5 in *D. citri* (Table 4). Unfortunately, the annotated models are missing a small amount of 3*′* sequence due to genome assembly issues. However, we identified a *de-novo* assembled transcript (MCOT06229.1.CO) that appears to encode the full-length protein (Figure 3). The missing genome sequence does not affect the conserved functional domains of CDA5. Four of the five transcript isoforms encode proteins containing both an N-terminal ChBD and a C-terminal catalytic domain, as seen in other insect CDA5 orthologs. The remaining isoform (CDA5-RB) differs at the 5*′* end and apparently lacks a ChBD-encoding region.

### Other chitin deacetylase groups

We did not find any group II or group V CDAs in the *D. citri* v3 genome (Figure 4). To our knowledge CDAs from these groups have not been found in any hemipteran insects examined to date [6], so their absence in *D. citri* was expected.

## Re-use potential

This manual curation was carried out as a part of the Diaphorina citri community annotation project [38] with a goal to annotate gene families related to immune response, metabolism and other major functions [39–42]. Our manual annotations will be useful for researchers studying the chitin deacetylase genes in the future. We annotated multiple isoforms for three of the four genes, which will inform the design of experiments to determine the expression pattern and function of specific isoforms. Our annotations will be incorporated into an updated official gene set and will be publicly available for comparative expression profiling on the CGEN [20].

## Data Availability

The *Diaphorina citri* genome assembly, gene sets, and transcriptome data are accessible via the Citrus Greening website [20]. All accessions for genes used for phylogenetic analysis are provided within this report, and all additional data is available via the *GigaScience* GigaDB repository [43].

## Editor’s Note

This article is one of a series of Data Releases crediting the outputs of a student-focused and community-driven manual annotation project curating gene models and if required, correcting assembly anomalies, for the *Diaphorina citri* genome project [13].

## List of abbreviations

CDA: chitin deacetylase; CGEN: Citrus Greening Expression Network; ChBD: chitin binding domain; *C*Las: *Candidatus* Liberibacter asiaticus ; NCBI: National Center for Biotechnology Information; RNAi: RNA interference; TPM: transcripts per million.
